# Low-Dose Aspirin and Progression of Age-Related Hearing Loss: A Secondary Analysis of the ASPREE Randomized Clinical Trial

**DOI:** 10.1001/jamanetworkopen.2024.24373

**Published:** 2024-07-25

**Authors:** David P. Q. Clark, Zhen Zhou, Sultana M. Hussain, Cammie Tran, Carlene Britt, Elsdon Storey, Judy A. Lowthian, Raj C. Shah, Harvey Dillon, Rory Wolfe, Robyn L. Woods, Gary Rance, John J. McNeil

**Affiliations:** 1 The School of Public Health and Preventive Medicine, Monash University, Victoria, Australia; 2 Department of Epidemiology and Preventive Medicine, Alfred Hospital, Victoria, Australia; 3 The School of Population and Global Health, The University of Melbourne, Victoria, Australia; 4 Bolton Clarke Research Institute, Forrest Hill, Victoria, Australia; 5 Department of Family and Preventive Medicine, Rush University Medical Center, Chicago, Illinois; 6 Rush Alzheimer’s Disease Center, Rush University Medical Center, Chicago, Illinois; 7 The HEARing Cooperative Research Centre, Melbourne, Victoria, Australia; 8 Hearing Research Centre, Macquarie University, New South Wales, Australia; 9 Manchester Centre for Audiology and Deafness, Faculty of Biology, Medicine and Health, University of Manchester, Manchester, United Kingdom

## Abstract

**Importance:**

Age-related hearing loss is common in an aging population, affecting communication and contributing to a worsened quality of life. It occurs as a result of cochlear degeneration and may be further exacerbated by inflammation and microvascular changes, as observed in animal models.

**Objective:**

To compare the effect of daily low-dose aspirin vs placebo on the progression of age-related hearing loss in healthy older adults.

**Design, Setting, and Participants:**

A prespecified secondary analysis was conducted of the Aspirin in Reducing Events in the Elderly (ASPREE) randomized clinical trial. Participants were 279 healthy community-dwelling individuals living in Australia who were aged 70 years or older and free of overt cardiovascular diseases, dementia, and life-limiting illnesses. Participants were recruited between January 1, 2010, and December 31, 2014, and followed up over 3 years. Statistical analysis was completed from June to December 2023.

**Intervention:**

A 100-mg daily dose of enteric-coated aspirin or matching placebo.

**Main Outcomes and Measures:**

Hearing measures were air conduction audiometry and binaural speech perception in noise. Assessments were conducted at baseline, 18 months, and 3 years. The change from baseline hearing measures were analyzed using an intention to treat approach. Aspirin and placebo were compared using mixed linear regression models adjusting for age, sex, diabetes, and smoking.

**Results:**

Of 279 participants, 154 (55%) were male, and the median age at baseline was 73.1 years (IQR, 71.5-76.2 years). A total of 98 of 138 participants (71%) in the aspirin group and 94 of 141 participants (67%) in the placebo group reported experiencing hearing loss at baseline. Compared with placebo, aspirin did not affect the changes in mean (SD) 4-frequency average hearing threshold from baseline to year 3 (aspirin: baseline, 27.8 [13.3] dB; year 3, 30.7 [13.7] dB; difference, 3.3 [3.9] dB; placebo: baseline, 27.5 [12.6] dB; year 3, 30.9 [13.8] dB; difference, 3.0 [4.8] dB; *P* = .55) nor any other tested frequencies. An increase in air conduction threshold indicates a deterioration in hearing. Similarly, for the mean (SD) speech reception threshold, there was no significant difference observed between the aspirin and placebo group at the year 3 follow-up assessment (aspirin: baseline, –9.9 [3.8] dB; year 3, –9.1 [3.8] dB; difference, 0.9 [2.9] dB; placebo: baseline, –10.5 [7.1] dB; year 3, –9.6 [4.1] dB; difference, 0.9 [5.9] dB; *P* = .86). The findings were consistent across sex, age groups, diabetic and smoking status.

**Conclusions and Relevance:**

In this secondary analysis of the ASPREE randomized clinical trial, low-dose aspirin did not affect the progression of age-related hearing loss. More investigation is warranted on whether a longer follow-up or the use of a more powerful anti-inflammatory agent might prove beneficial.

**Trial Registration:**

anzctr.org.au Identifier: ACTRN12614000496617

## Key Points

**Question:**

Does daily low-dose aspirin slow the progression of age-related hearing loss in healthy older adults aged 70 years or older?

**Findings:**

In this prespecified secondary analysis comprising 279 participants aged 70 years or older in the randomized ASPREE clinical trial, aspirin use did not affect the age-related decline in hearing threshold or in binaural speech perception threshold compared with placebo over a follow-up period of 3 years.

**Meaning:**

This study suggests that, in healthy older adults, low-dose aspirin does not affect the progression of age-related hearing loss.

## Teaser

This prespecified secondary analysis of the ASPREE randomized clinical trial compares the effect of daily low-dose aspirin vs placebo on the progression of age-related hearing loss in healthy older adults.

## Introduction

Age-related hearing loss is estimated to affect over half of adults aged 70 years or older and is associated with social isolation, depression, loss of functional independence, and poor quality of life.^1,2,3,4^ It is characterized by impaired speech discrimination and progressive, bilateral, high frequency loss, observed on audiometric assessment. Established risk factors for age-related hearing loss include smoking and diabetes^5,6^

The pathophysiology of age-related hearing loss is thought to involve an age-related decline in cochlear function accompanied by degenerative changes in central auditory pathways.^7^ Within the cochlea, the predominant pathology includes atrophy of the stria vascularis, together with a loss of hair cells and synaptic connections among spiral ganglion neurons.^8^ Animal studies have provided evidence of microvascular changes, including capillary loss, which may contribute to strial atrophy.^9^

Aspirin has been investigated on account of its ability to prevent platelet aggregation and potentially enhance blood flow through small striatal blood vessels.^10^ In addition, its anti-inflammatory effect may reduce damage at a cellular level.^11^ To date, relevant human data on aspirin have been derived from observational studies and the results have been inconsistent.^12,13^ A review in 2014 summarized 37 of these studies and noted poorer hearing outcome among individuals taking more than 1.95 g/d of aspirin, but no studies focused on low-dose aspirin.^14^ We have not identified any randomized clinical trial comparing the effect of low-dose aspirin vs placebo on the progression of age-related hearing loss.

The ASPREE-Hearing (Aspirin in Hearing, Retinal Vessels Imaging and Neurocognition in Older Generations) substudy of the ASPREE (Aspirin in Reducing Events in the Elderly) randomized clinical trial offers a unique opportunity to address inconsistencies reported among previous observational studies. Identification of an effective approach to reduce age-related hearing loss progression might reduce the burden of cost and morbidity associated with this prevalent sensory disturbance. We aimed to determine whether daily low-dose aspirin slows age-related hearing loss progression in healthy community-dwelling older adults.

## Methods

### Trial Design and Setting

The ASPREE-Hearing substudy is embedded within the parent ASPREE clinical trial, details of which have been previously published.^15^ In brief, ASPREE was a double-blinded, randomized placebo-controlled trial that aimed to assess the effect of low-dose aspirin on disability-free survival in relatively healthy people aged 70 years or older (or ≥65 years for US participants of racial and ethnic minority groups). A total of 19 114 participants were recruited between January 1, 2010, and December 31, 2014. At entry, all were free of overt cardiovascular disease, dementia, significant physical disability, or any illness expected to limit their life expectancy to 5 years or less. Participants were followed up face to face annually and by telephone every 6 months. Baseline data collection included anthropometric and laboratory test measurements, as well as questionnaires concerning comorbidities and a social and medical history. Written consent was obtained from all participants. Ethics approval was provided by the Monash University human research ethics committee for both the ASPREE parent trial and the ASPREE-Hearing substudy. This prespecified secondary analysis was reported according to the Consolidated Standards of Reporting Trials (CONSORT) reporting guideline.

### Intervention and Randomization Procedures

Eligible participants underwent a 4-week placebo run-in phase to assess adherence. Those achieving 80% or more adherence were then randomized to receive either 100 mg daily of enteric-coated aspirin or matching placebo, in a 1:1 ratio, using a block-randomization procedure. Participants and study staff were blinded to group allocation.

### ASPREE-Hearing Substudy

Newly recruited Australian ASPREE participants in 2014 were invited to participate in the ASPREE-Hearing substudy. Details are described in the trial protocol and statistical analysis plan in Supplement 1.^16^ Participants with bilateral cochlear implants or bilateral deeply inserted per-tympanic or implanted hearing aids were excluded. Individuals using other hearing devices were not excluded.

Hearing assessments were conducted in medical clinic rooms, community centers, or purpose-built mobile vans following a standard operating procedure.^17^ Sound-attenuating earmuffs were used across all testing environments with any hearing aids removed. An otoscopic examination was performed at each hearing visit and, in the case of occlusion of the auditory canal by wax, hearing from the unobstructed ear was measured. Participants with bilateral obstruction were advised to consult their family physician for management before reexamination. Hearing assessments occurred at baseline and at 18 months and 3 years after randomization.

### Hearing Tests

Air conduction measures were undertaken using portable audiometers (AD226; Interacoustics A/S) with ER3A fitted inserts and sound-attenuating earmuffs (Australian Standards AS/NZS 1270:2002). Sound detection thresholds were determined at 0.25-, 0.5-, 1-, 2-, 4-, and 8-kHz pure tones for each ear. A mean of sound conduction thresholds at 0.5, 1, 2, and 4 kHz in the better ear (termed *4FA*) reflects the frequency range relevant to speech audibility. All hearing thresholds were measured in decibels.

Hearing impairment categories were classified using the World Health Organization hearing impairment grading system.^18^ Normal hearing refers to a hearing threshold less than 20 dB, while a higher (or increase in) audiometric threshold indicates worse (or worsening) hearing.

The Listening in Spatialized Noise–Sentences Test, which assesses the ability to hear and understand speech in the presence of background noise, was also administered at each follow-up.^19^ Headphones were used to replicate an “everyday” listening situation with target sentences and background noise spatially separated by 90° and the volume of the speech stimuli adjusted according to each participant’s hearing acuity. Results are shown as the speech reception threshold (SRT)—by convention referred to as a *signal to noise ratio*—but in fact is a difference level (ie, the sound pressure level difference [dB] between the target speech signal and the background noise) required for the listener to identify 50% of the target words in a series of test sentences. Better performance is indicated by a more negative number. Young adults with normal hearing achieve this performance, on average, when the difference between the signal and the noise is of the order of −17.4 dB, meaning that they can identify half the target words when the noise is 17.4 dB louder than the target.

The 10-item Hearing Handicap Inventory for the Elderly (HHIE-S)^20^ and 11-item Baltimore Longitudinal Study of Aging Self-Reported Hearing and Noise Exposure Questionnaire^21^ were also used to collect information on hearing history, noise exposure, use of hearing aids, and perceived hearing handicap.

### Outcomes

The main outcome of this study was hearing measures, defined as the mean sound detection thresholds for individual pure tones, 4FA, and SRT, measured at baseline, 18 months, and 3 years. The parent trial was stopped prematurely in June 2017 at the request of the funders. This decision came after the comparable incident rates of the primary end point in the aspirin and placebo groups showed that the continuation of the trial medication until its scheduled end date of December 31, 2017, would unlikely identify any treatment benefit. Consequently, only 22% of the ASPREE-Hearing substudy participants (279 of 1269) had their year 3 audiometry assessment completed within the prescribed randomization period, and they were included in the primary analysis.

### Statistical Analysis

The statistical analysis was performed from June to December 2023. Baseline summary statistics included mean (SD) values for continuous variables with normal distribution, median (IQR) values for skewed continuous variables, and number (percentage) for categorical variables. The outcome analysis is based on intention to treat.

A linear mixed model was used to compare changes in hearing measures between the treatment groups, from baseline to the year 3 follow-up. The model included fixed effects of treatment group, time (baseline, 18 months, and 36 months), and treatment × time interaction; random slopes and random intercepts were included to account for interindividual variability. The estimated interaction effect at year 3 provided evidence for a population-average effect. The linear mixed model assumes a missing-at-random mechanism for unobserved hearing values.

In a subsequent secondary analysis, the same model structure was used with further adjustments for the covariates of age, sex, smoking, and diabetes. These covariates were prespecified in the trial protocol^16^ and were included to potentially improve the precision of the fitted model. Further analyses by the sound detection thresholds for the 4FA and the 4-kHz frequency were performed to assess speech intelligibility and high-frequency hearing loss in older adults. Exploratory subgroup analysis by age, sex, smoking, and diabetes were undertaken for the outcomes of 4FA, the high-frequency 4 kHz, and SRT. Modification of the effect of aspirin by the covariates was assessed with the use of a 3-way interaction term (stratifying variable × aspirin treatment × time).

A sensitivity analysis was performed to include the participants whose 3-year examination occurred at 3 or 6 months after randomization. This analysis takes into account a likely short-term continuation of aspirin effect after discontinuation of active treatment, allowing a larger number of randomized participants to be included in the analysis. A 2-sided *P* < .05 was used as a cutoff for statistical significance for all analyses, including subgroup analysis without adjustment for multiple comparisons. All statistical analyses were performed using Stata, version 17 (StataCorp LLC).

## Results

**Figure 1.**
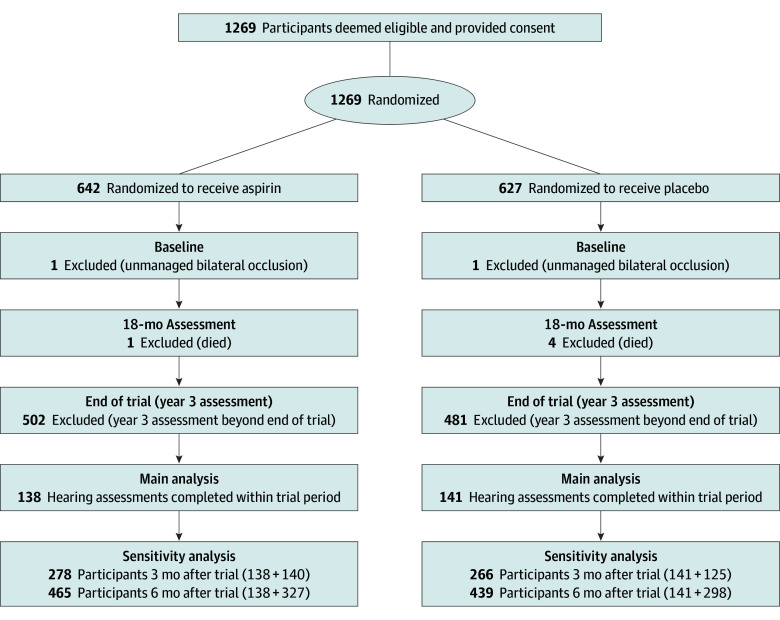
Trial Flow Diagram

The flow diagram of the study is shown in Figure 1. After excluding 2 people with unmanaged bilateral occlusion at baseline, 5 who died prior to the 18-month follow-up, and 983 individuals who failed to complete their third hearing visit within the prescribed trial period, the final analysis included 279 participants: 138 assigned to aspirin and 141 assigned to placebo.

Table 1 shows the baseline characteristics, which were well-matched between the randomized groups. Definitions for covariates are outlined in the eAppendix in Supplement 2. The median age was 73.1 years (IQR, 71.5-76.2 years), with 154 men (55%) and 125 women (45%). Approximately 70% in each group had some hearing loss at baseline (71% [98 of 138] in the aspirin group and 67% [94 of 141] in the placebo group). In addition, 17% of participants (23 of 138) in the aspirin group and 19% of participants (27 of 141) in the placebo group reported using hearing aids, and 17% of participants (23 of 138) in the aspirin group and 8% of participants (11 of 141) in the placebo group reported a history of loud noise exposure (at work and outside of work) in the previous 5 years. Similar proportions in both groups were never smokers (aspirin, 56% [77 of 138]; placebo, 57% [80 of 141]) or never consumed alcohol (aspirin, 15% [21 of 138]; placebo, 13% [18 of 141]). At baseline, most participants were nonfrail^22^ (aspirin, 71% [98 of 138]; placebo, 68% [96 of 141]) and free from diabetes (aspirin, 89% [123 of 138]; placebo, 86% [121 of 141]). Hypertension was reported by 70% of participants (97 of 138) in the aspirin group and by 72% of participants (101 of 141) in the placebo group. Study medication adherence in the parent ASPREE trial, measured by pill count, assessed during the 6-month follow-up telephone call, was approximately 70% for both groups.^23^ When the baseline characteristics of participants included in the primary analysis were compared with those excluded, all covariates were similar except for a higher proportion of women among those excluded (54% [533 of 983] vs 45% [125 of 279]) (eTable 3 in Supplement 2).

**Table 1.** Baseline Characteristics of Participants, Stratified by Treatment Group

Characteristic^a^	Total participants, No. (%) (N = 279)	Placebo, No. (%) (n = 141)	Aspirin, No. (%) (n = 138)
Age			
≤75 y	188 (67)	92 (65)	96 (70)
>75 y	91 (33)	49 (35)	42 (30)
Sex			
Male	154 (55)	77 (55)	77 (56)
Female	125 (45)	64 (45)	61 (44)
Alcohol use			
Current or former	240 (86)	123 (87)	117 (85)
Never	39 (14)	18 (13)	21 (15)
Smoking			
Current or former	122 (44)	61 (43)	61 (44)
Never	157 (56)	80 (57)	77 (56)
Frailty			
Not frail	194 (70)	96 (68)	98 (71)
Prefrail or frail	85 (30)	45 (32)	40 (29)
Diabetes			
Yes	35 (13)	20 (14)	15 (11)
No	244 (87)	121 (86)	123 (89)
eGFR			
<45 mL/min/1.73 m^2^	5 (2)	4 (3)	1 (1)
≥45 mL/min/1.73 m^2^	274 (98)	137 (97)	137 (99)
Hypertension			
Yes	198 (71)	101 (72)	97 (70)
No	81 (29)	40 (28)	41 (30)
Hearing aid use			
Yes	50 (18)	27 (19)	23 (17)
No	229 (82)	114 (81)	115 (83)
Hearing loss severity			
Normal (<20 dB)	86 (31)	46 (33)	40 (29)
Mild (20-34 dB)	117 (42)	55 (38)	62 (45)
Moderate (35-49 dB)	58 (21)	29 (21)	29 (21)
Moderately severe (50-64 dB)	14 (5)	8 (6)	6 (4)
Severe to profound (≥65 dB)	2 (1)	1 (1)	1 (1)
Loud noise exposure			
Yes	34 (12)	11 (8)	23 (17)
No	241 (88)	129 (92)	112 (83)

Abbreviation: eGFR, estimated glomerular filtration rate.

^a^ Covariate measures: Diabetes is defined from self-report or fasting glucose of 126 mg/dL or more (to convert to mmol/L, multiply by 0.0555) or taking glucose-lowering medications. Hypertension is defined as blood pressure of 140/90 mm Hg or higher or taking antihypertensive medications. “Frail” included individuals meeting 3 or more criteria of the adapted Fried frailty criteria, including body weight, strength, exhaustion, walking speed, and physical activity, while “prefrail” was defined as meeting 1 or 2 criteria.^22^ For measurement of fasting blood glucose, participants were required to fast overnight, and their blood samples were collected in a local clinic or pathology center. Blood pressure for each participant was measured in the seated position after at least 5 minutes of rest using an automated oscillometric device with an occluding cuff of appropriate size for the upper arm circumference. Three separate and consecutive blood pressure readings, 1 minute apart, were performed, and the mean of these measurements was recorded. Other variables were collected by questionnaire.

### Hearing Acuity and Progression Over 3 Years

**Figure 2.**
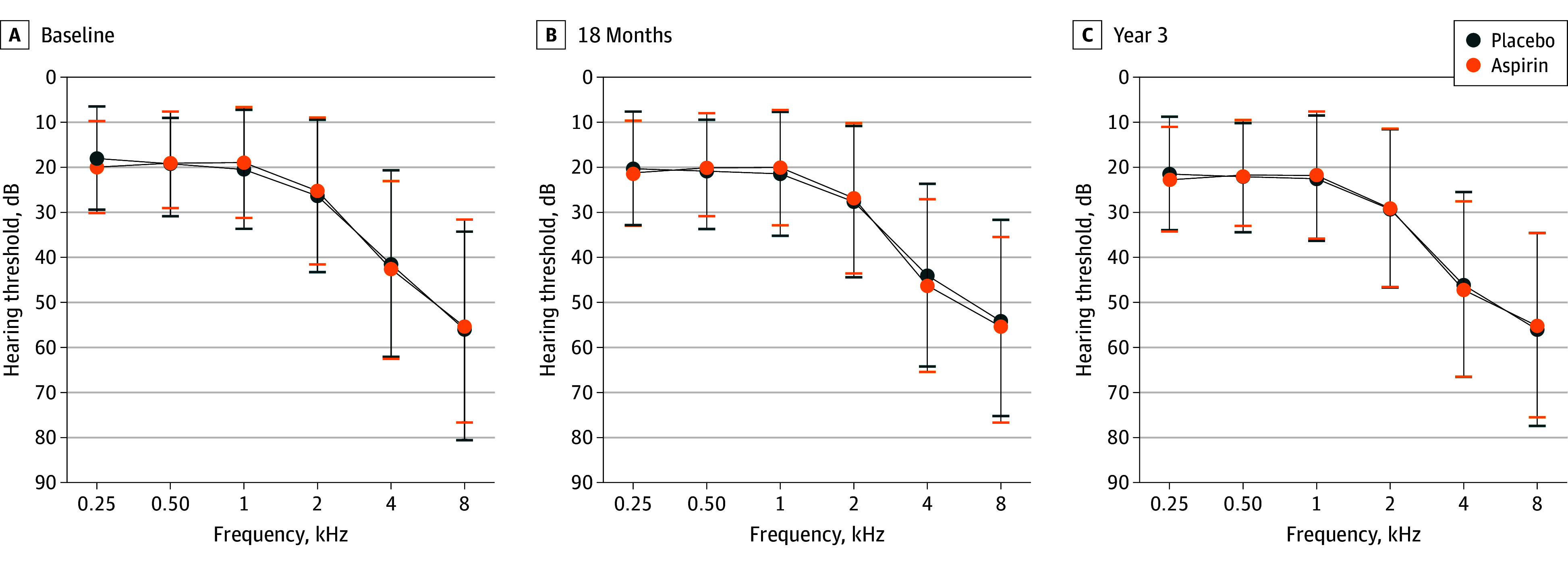
Mean Hearing Thresholds for Pure Tone Audiometry in Aspirin and Placebo Groups Error bars indicate SD.

Mean (SD) hearing acuity at baseline, 18 months, and year 3 follow-up visits are shown in Figure 2 and Table 2. Although the mean 4FA sound detection thresholds increased over the 3 years (from 27.5 [12.6] dB to 30.9 [13.8] dB in the placebo group [difference, 3.0 (4.8) dB] and from 27.8 [13.3] dB to 30.7 [13.7] dB in the aspirin group [difference, 3.3 (3.9) dB]), the progression of hearing acuity from baseline to year 3 did not differ between the groups for any of the tested pure tones.

**Table 2.** Mean Hearing Thresholds and the Proportions of the Same Participants at Each Pure Tone, Stratified by Treatment Groups

Hearing measure	Baseline		18 mo		Year 3	
	Placebo (n = 141)	Aspirin (n = 138)	Placebo (n = 141)	Aspirin (n = 138)	Placebo (n = 141)	Aspirin (n = 138)
Sound frequency, mean (SD), kHz						
0.25	18.1 (11.4)	20.0 (10.2)	20.4 (12.7)	21.3 (11.6)	21.4 (12.6)	22.7 (11.6)
0.5	19.3 (11.6)	19.1 (10.0)	20.9 (12.9)	20.2 (10.7)	22.0 (12.4)	21.6 (11.4)
1	20.5 (13.2)	19 (12.3)	21.5 (13.8)	20.1 (12.8)	22.4 (13.9)	21.7 (14.1)
2	26.4 (16.9)	25.3 (16.3)	27.7 (16.8)	26.9 (16.7)	29.2 (17.6)	29.0 (17.6)
4	41.6 (20.9)	42.6 (19.5)	44.0 (20.3)	46.3 (19.2)	46.0 (20.5)	47.1 (19.5)
8	56.0 (24.4)	55.4 (21.1)	54.2 (22.5)	55.4 (19.9)	55.9 (21.5)	55.1 (20.4)
4FA	27.5 (12.6)	27.8 (13.3)	29.4 (13.3)	29.4 (12.7)	30.9 (13.8)	30.7 (13.7)
SRT, mean (SD), dB	−10.5 (7.1)	−9.9 (3.8)	−9.2 (4.1)	−8.6 (4.4)	−9.6 (4.1)	−9.1 (3.8)
HHIE-S, No. (%)						
No handicap	96 (69)	89 (66)	85 (62)	84 (62)	86 (61)	87 (63)
Mild-moderate handicap	34 (24)	39 (29)	44 (32)	42 (31)	46 (33)	40 (29)
Severe handicap	10 (7)	7 (5)	9 (6)	10 (7)	9 (6)	11 (8)

Abbreviations: 4FA, mean of pure tones at 0.5, 1, 2, and 4 kHz; HHIE-S, Hearing Handicap Inventory- for the Elderly Screening Version (score 0-8, no handicapped; score 10-24, mild-moderate handicap; score 26-40, severe handicap [each item answered in the questionnaire is awarded a score of 2; the sum of all the items can only be in increments of 2]); SRT, speech reception threshold.

Similarly, there were no significant differences in the mean (SD) SRT at baseline, 18 months, or year 3 between aspirin and placebo (from −10.5 [7.1] dB to −9.6 [4.1] dB in the placebo group [difference, 0.9 (5.9) dB] and from −9.9 [3.8] dB to −9.1 [3.8] dB in the aspirin group [difference, 0.9 [2.9] dB]) (Figure 2 and Table 2). At baseline, self-reported hearing handicap, using the HHIE-S criteria, was 31% (44 of 140) for the placebo group and 34% (46 of 135) for the aspirin group. By year 3, there was a modest increase in the prevalence of self-reported hearing handicap; however, the proportions remained similar (39% [55 of 141] for placebo and 37% [51 of 138] for aspirin).

**Figure 3.**
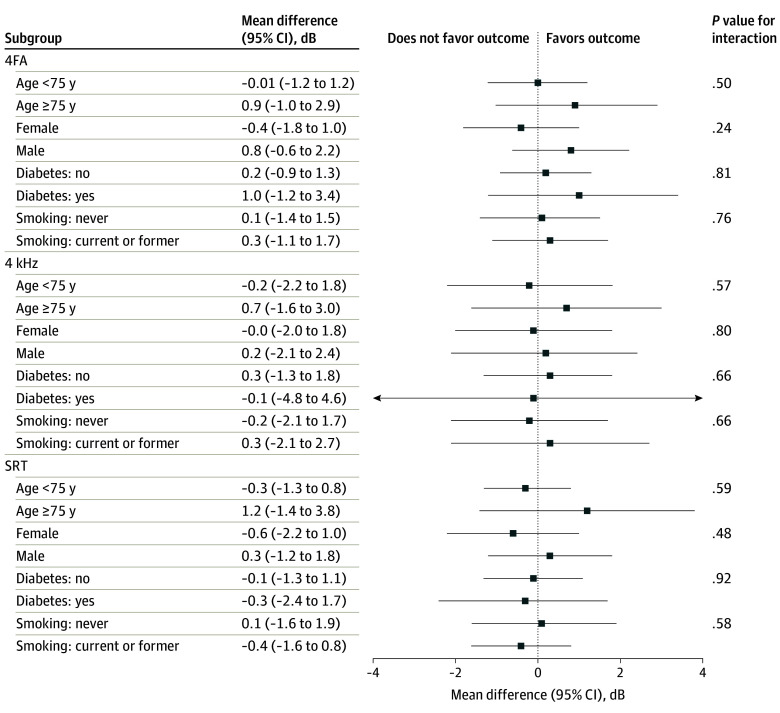
Subgroup Analysis: Modification by Prespecified Variables on the Effect of Aspirin vs Placebo on the Changes in Mean Hearing Thresholds Between Baseline and Year 3 4FA indicates mean of pure tones at 0.5, 1, 2, and 4 kHz; SRT, speech reception threshold.

The forest plots in Figure 3, stratified by age (>75 or ≤75 years), sex, diabetes, and smoking status, showed no modification effect by treatment on the change in hearing measures from baseline to year 3, either for the 4FA, 4 kHz, or for the SRT analysis.

eTable 1 in Supplement 2 provides the difference in changes in mean hearing thresholds between baseline and year 3, comparing the aspirin group with the placebo group. The changes in mean 4FA and other pure tones tested were similar in both groups. The mean difference in change for 4FA was 0.31 dB (95% CI, –0.72 to 1.34 dB). Similarly for SRT, there was no statistically significant difference when the treatment groups were compared (mean difference in change, −0.1 [95% CI, –1.2 to 1.0]; *P* = .86). After further adjustment for age, sex, diabetes, and smoking, the results did not differ from those of the unadjusted analysis.

### Sensitivity Analysis

A total of 544 participants (278 aspirin, 266 placebo) completed their year 3 hearing assessment within 3 months after the trial, whereas 904 participants (465 aspirin, 439 placebo) completed within 6 months after the trial (Figure 1). These participants were added back into the main analysis (eTable 2 in Supplement 2). The results revealed no differences between the treatment groups for the change in hearing threshold at 4 kHz, the 4FA, or the SRT from baseline to either 3 or 6 months after the trial assessment.

## Discussion

This study has demonstrated that an intervention using 100 mg of aspirin daily over a 3-year follow-up had no discernible effect on the progression of hearing loss among healthy participants aged 70 years or older. The result was unchanged when participants were stratified according to age (≤75 and >75 years), sex, among smokers and ex-smokers, and individuals with diabetes. Low-dose aspirin also did not affect SRT, an indicator of functional hearing ability, or perceived functional limitations associated with hearing impairment (HHIE-S).

The rationale for studying the effect of aspirin was based on its potential to affect key aspects of the pathology of age-related hearing loss. There is evidence that degenerative changes affecting the microcirculation of the cochlea may be important in the development of age-related hearing loss.^24,25^ Strial atrophy secondary to strial microvascular degeneration has been demonstrated in mouse models, while strial capillary loss has been strongly associated with presbycusis in aged gerbils.^7,26^ Capillary histologic findings in the presbycusis-gerbils models have revealed thickened basement membranes and vascular deposits of laminin and immunoglobulins.^27^ The potential of aspirin, through its ability to prevent platelet aggregation and clumping, to maintain blood circulation through aging capillaries provides a rationale to explore its action in delaying the progress of age-related hearing loss.

Another potentially useful property of aspirin is its anti-inflammatory action. Chronic low-grade inflammation accompanies aging in most animal species and has been implicated in age-related hearing loss. Elevated levels of inflammatory cytokines such as tumor necrosis factor may contribute to microvascular changes.^28^ In a population-based cohort study, older individuals with higher levels of C-reactive protein were twice as likely as those with lower levels to develop hearing loss.^29^ Aspirin has been reported to inhibit inflammatory mediators and promote the synthesis of anti-inflammatory compounds, providing another rationale to investigate a protective effect on hearing.^30,31^

Two previous double-blind randomized trials have focused on high doses of aspirin for the prevention of gentamycin-induced ototoxicity.^32,33^ Both trials produced inconsistent results and have minimal relevance to a long-term preventive setting. The most significant observational data were from the US Health Professionals longitudinal study analyzing analgesic use (including aspirin) in relation to hearing impairment.^12^ Regular intake of aspirin was associated with an increased risk of self-reported hearing impairment (hazard ratio, 1.33; 95% CI, 1.03-1.72). To our knowledge, no prospective randomized trials have reported the effect of chronic low-dose aspirin therapy on the progress of age-related hearing loss.

During the present trial, a gradual deterioration in hearing acuity was observed in our study population, with the threshold values of pure-tone audiometry increasing by approximately 3 dB over a 3-year period. This change aligns with previous studies reporting that after 60 years of age, hearing thresholds tend to worsen, with a mean decrease of 1 dB per year.^34,35^ The results also demonstrated a more rapid decrease in hearing sensitivity observed at higher pure tones (4 and 8 kHz), where the effects of aging are most prominent.^36^ The period of aspirin administration therefore corresponded with a measurable deterioration in hearing acuity in both the aspirin and placebo group.

Similarly, the change in SRT during the intervention period was also evident in both treatment groups, reflecting a decrease in an individual’s capacity to hear and communicate in everyday listening situations. The clinical significance of the mean 0.9-dB change in SRT over the 3-year study period can be interpreted in the context of a 1-dB decrease in SRT corresponding to a reported a 14% to 19% reduction in speech intelligibility.^37^ As such, the study participants in both the aspirin and placebo groups experienced a substantial but virtually identical decrease in functional hearing ability over the course of the study.

The clinical implications of this study result from the widespread regular use of aspirin among older individuals and the observational data linking chronic use of analgesic use with hearing loss. Despite the lack of an expected beneficial effect of low-dose aspirin, we did not identify a deleterious effect similar to that seen in the US Health Professionals observational study.^12^

### Strengths and Limitations

This study has some strengths, including the randomized double-blinded design and the selection of generally healthy community-dwelling participants who were free from the confounding influence of other chronic illnesses. The objective measurement of hearing and similar results from both the auditory acuity and the speech perception in noise test, which is particularly sensitive to auditory dysfunction at older ages, also contributes to the validity of the results. Results are mainly generalized to a relatively healthy White population living in Australia.

The study also has some limitations, including the small sample size and relatively modest duration of follow-up, which do not preclude the possibility of a different result with a longer treatment phase. Both were partially addressed by lack of efficacy of aspirin seen in the sensitivity analyses, which included participants who had received over 30 months of randomized treatment but were not followed up until 3 or 6 months after the closure of the trial phase. Our principal outcome measure relied on pure-tone audiometry, which is not an objective measure because it requires volitional responses to auditory stimuli. Nonetheless, it is the criterion standard for hearing level assessment and consistently shows high retest reliability in cooperative and attentive adults.^38^ Although there is evidence to suggest slight reliability decreases in older adults (perhaps related to anatomical changes in the aging ear canal or attention changes), retest reliability estimates of less than 5 dB have been reported for individuals up to the age of 80 years.^39^ Despite the lack of an observable effect of low-dose aspirin in our study, the results do not exclude the possibility that more prolonged treatment or the use of a more powerful anti-inflammatory agent might prove beneficial.

## Conclusions

This secondary analysis of a randomized clinical trial of a daily 100-mg low dose of aspirin over 3 years did not demonstrate any significant effect on the progression of age-related hearing loss. Although our findings add valuable data to the ongoing discussion surrounding aspirin and hearing health, future trials will be necessary to determine whether other anti-inflammatory or antiplatelet agents exert a protective effect on hearing loss. Furthermore, the complex relationship between aspirin, inflammation, and hearing loss warrants continued investigation to elucidate potential mechanisms and clinical implications.

## Notes

Supplement 1.
Trial Protocol and Statistical Analysis Plan

Supplement 2. **eTable 1.** Changes in Mean Hearing Thresholds (dB) Between Baseline and Year 3, and Comparing Aspirin to Placebo Groups **eTable 2.** Sensitivity Analysis—Difference in Change in Mean Hearing Thresholds (dB) Between Baseline and Year 3, Comparing Aspirin to Placebo Groups **eTable 3.** Demographic Characteristics Comparing Those Included in the Main Analysis and Those Excluded Due to Their Year 3 Hearing Evaluation Being Conducted After the Trial Period Concluded **eAppendix.** Covariate Measures
eReference

Supplement 3.
Data Sharing Statement
