# Supplementary material for: Low-Dose Aspirin and Progression of Age-Related Hearing Loss: A Secondary Analysis of the ASPREE Randomized Clinical Trial
Source: JAMA Netw Open. 2024 Jul 25;7(7):e2424373. doi: 10.1001/jamanetworkopen.2024.24373 (PMC11273233; doi:10.1001/jamanetworkopen.2024.24373)
Supplement: Supplement 1. — Trial Protocol and Statistical Analysis Plan [file jamanetwopen-e2424373-s001.pdf]

ASP-HEARING: LOW-DOSE ASPIRIN AND AGE-RELATED HEARING LOSS, A RANDOMISED CONTROLLED TRIAL

INTRODUCTION

Hearing loss is one of the leading causes of disease burden in older Australians, and is associated with poorer health, loss of functional independence, communication difficulties, social isolation, poor quality of life, falls, hospitalisation and all-cause mortality. (1) The Australian burden of presbyacusis, or age-related hearing loss (ARHL), is substantial, with a majority of those over 70 years commonly afflicted. Targeting the national decline in hearing in this age group will have a significant impact on quality of life of older Australians while addressing a profound public health issue. Access Economics estimated the financial burden of hearing loss in 2005 at $3,314 per person with hearing loss, $578 per Australian and 1.4% of GDP (2). The Australian government identifies hearing loss as a key health prevention area.

Clinical characteristics of ARHL include bilateral, symmetrical loss of threshold sensitivity in the high frequency region of the hearing spectrum, with rapid advancement after age 70 years. It is the most common cause of hearing loss in developed countries.

Hearing loss prevention is an important goal for health systems, health professionals and individual sufferers. Public health approaches have been limited to modifying the disability and handicap of hearing loss through rehabilitation and electronic device support. Although Australia has successfully campaigned for prevention of noise related hearing loss through workplace ear protection, there is currently no clear approach to the prevention of the more significant age-related hearing loss, presbyacusis. To date, the search for modifiable determinants (3) and pharmacotherapies has been relatively disappointing.

ASP-HEARING, (**ASP**irin in **HEA**ring, **R**etinal vessels, **I**nflammatory markers and **N**euro-cognition in older age **G**roups) is a three year randomised controlled trial of low dose aspirin versus placebo, that will investigate whether low dose aspirin has potential as a cost-effective, simple, primary care intervention to slow the progression of ARHL.

There is some uncertainty about the aetiology of ARHL, but we propose testing a feasible inflammatory and microvascular mechanism using an intervention with low dose aspirin while recording changes in hearing thresholds, speech reception thresholds, biomarker levels and retinal blood vessels. Additionally, as cognitive decline has been observed as an independent associate of ARHL, after controlling for age, sex, race, education, diabetes, smoking and hypertension (4) our study will interrogate the relationship between hearing loss and cognitive decline.

AIMS AND HYPOTHESES

Main aim: To determine whether low dose aspirin decreases progression of age-related hearing loss.

Main hypothesis: Low dose aspirin will reduce progression of age-related hearing loss.

The basis of this hypothesis is that aspirin is an anti-inflammatory agent, which even in low dose may produce significant suppression of plasma biomarkers of inflammation. If a low-grade inflammatory process, either directly or via microvascular change, contributes to the pathogenesis of ARHL, then aspirin will slow the progression of hearing loss.

Subsidiary hypotheses:

1) **Hearing threshold will correlate with inflammatory biomarkers, Tumor Necrosis Factor-alpha(TNF-α), InterLeukin-6 (IL-6) or C-Reactive Protein (CRP)**
- Hearing threshold at baseline will correlate with levels of inflammatory biomarkers at baseline.
- The trajectory of change in hearing threshold from baseline through to Year 3 will correlate with levels or change from baseline of inflammatory biomarkers at Year 3.
- Slowing of the rate of change in hearing threshold in the aspirin group will be associated with reduction of key inflammatory biomarkers.

The basis of this hypothesis is that an increase in plasma inflammatory biomarkers regularly accompanies old-age, presumably reflecting low-grade inflammation. With increasing age, the stria vascularis of the middle ear undergoes changes compatible with damage from an inflammatory and/or vascular degenerative process.

2) Hearing threshold will correlate with retinal microvascular changes.

- Hearing threshold at baseline will correlate with the extent of retinal microvascular changes at baseline.
- The trajectory of change in hearing threshold from baseline through to Year 3 will correlate with progression of retinal microvascular changes across the study.
- Slowing of rate of change in hearing threshold in the aspirin group will be associated with slower progression of retinal microvascular changes.

The basis of this hypothesis is that age-related changes in the microvasculature in the stria vascularis may be a primary cause of ARHL. The retina enables quantitative imaging of the microvasculature. Such changes may then reflect vascular changes in the cochlea.

3) Hearing threshold will correlate inversely with cognitive function.

- Hearing threshold at baseline will correlate inversely with cognitive function at baseline.
- The trajectory of change in hearing threshold from baseline through to Year 3 will correlate with the trajectory of cognitive change across the study.
- Slowing of rate of change in hearing threshold in the aspirin group will be associated with greater preservation of cognitive function.

The basis of this hypothesis is that age related decreases in cognition and hearing may share a common pathogenesis, possibly due to low-grade inflammation and/or microvascular change. If so, it might be expected that both cognition and hearing would decline in parallel. Furthermore, if hearing loss directly contributes to cognitive decline via reduction of stimuli including social interaction, both might also decline in parallel.

BACKGROUND

HEARING LOSS IS HIGHLY PREVALENT IN OLDER ADULTS

ARHL takes the form of a progressive, bilateral, high frequency loss that is manifested on audiometric assessment of hearing threshold by a moderately down-sloping pure tone audiogram. (Fig. 1) This contrasts with the pattern resulting from noise exposure; which takes the form of a steeply sloping high frequency loss, often with a notch or dip in the 4kHz region, due to loss of outer hair cells in the basal cochlea.

The World Health Organisation (WHO) defines mild hearing impairment as >25dB Hearing Level (HL) in the better ear, after averaging across the sound-frequencies of 0.5, 1, 2, 4 kHz while >40dB HL is considered moderate, causing significant handicap.

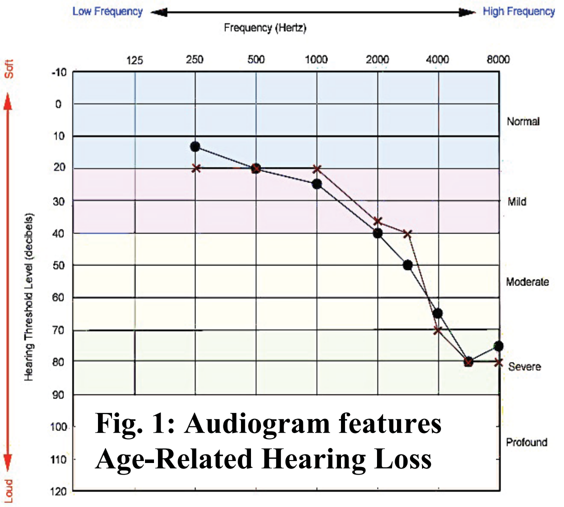

Fig. 1: Audiogram features Age-Related Hearing Loss

A large US study demonstrating hearing loss prevalence, showed that 45% of people have a HL of >25dB in their better ear in the 70-74 year age group, but 67% suffer ARHL in the 75-79 year group.(5, 6) In Australia the overall prevalence of hearing loss is similar for persons over 70 years (2, 7) and comparable per 5 year age brackets. (8) Hearing loss has far-reaching effects on an older adult's quality of life,(9) resulting in communication difficulties, social withdrawal, embarrassment, reduced self-esteem and depression. (10) Hearing loss is also associated with significant functional decline, cognitive impairment(4, 11), impaired activities of daily living (12), falls (13),

hospitalizations (14) and all-cause mortality.(1) Increased levels of the biomarkers CRP and IL-6 are linked with ARHL (15), and some genotypes are considered significant risk factors, such as APOE ε4. (16) Key messages from the epidemiological literature on ARHL are that there is no single predominant cause yet identified; and risk factors best established include: age, male gender, race, noise exposure, smoking, and diabetes.(17)

POTENTIAL ROLE OF INFLAMMATION IN AGEING AND HEARING LOSS

There is evidence that low-grade inflammation is a regular accompaniment of several common sequelae of ageing including cardiovascular disease, diabetes, cancer, frailty, mobility disability, fractures and cognitive decline. Plasma levels of a series of inflammatory markers have been shown to rise with age in apparently healthy individuals free of acute infection, including TNF-α, IL-6, and IL-1ra. (18) Plasma levels of CRP and white blood cell counts also increase with advancing years (19). Other key mediators of inflammatory reactions (*i.e.* IL-1β, COX-2, interferons and iNOS) have been shown to be up-regulated during the ageing process.

It is not clear whether inflammation is simply a reaction to an underlying pathological process, measure of the total chronic disease burden, or whether it contributes to the pathogenesis of multiple age-related diseases. An understanding of the relationship between low-grade inflammation and ageing may assist in revealing a basic mechanism of ageing, supporting the development of interventions that may promote healthy longevity. Insight must come from an intervention study demonstrating that reversal of the inflammatory processes is accompanied by mitigation of clinical symptoms.

Inflammation may play a fundamental role in hearing loss, yet current research is limited. In *animal* studies, IL-6, IL-1β and TNF-α are all significantly induced in noise exposed rodent cochleae. IL-6 expression is predominantly in the lateral wall of the spiral ligament and stria vascularis, where there is also evidence for up-regulation of IL-6 RNA with noise injury. (20) The histological findings suggest lateral wall fibrocytes as the main source of local inflammatory response to cochlear stress. Animal models of cisplatin-induced cytotoxicity also illustrate increases of pro inflammatory cytokines IL-6, IL-1β and TNF-α. (21) A mammalian model of presbyacusis, the senescence accelerated mouse, (SAMP8) was shown by Menardo *et al*, (22) to suffer high cochlear oxidative stress, and concomitant increase in TNF-α and IL-1β, suggesting that chronic inflammation is a causal factor for premature ARHL in mice.

In humans, a cross-sectional analysis of 611 older adults within the Hertford Aging Study cohort, showed that four measures of inflammatory status were associated with hearing loss. The white blood cell count, neutrophil count and inflammatory biomarkers IL-6 and CRP were significantly associated with worse average hearing threshold in older people, after adjustment for the effects of gender, smoking status and noise exposure. (15) The Epidemiology of Hearing Loss Study of 1073 participants, demonstrated that those aged <60 years with consistently high CRP or increasing levels of CRP over 10 years, were nearly twice as likely to develop hearing impairment over the 10 year period. (23)

POTENTIAL ROLE OF MICROVASCULAR DISEASE IN HEARING LOSS

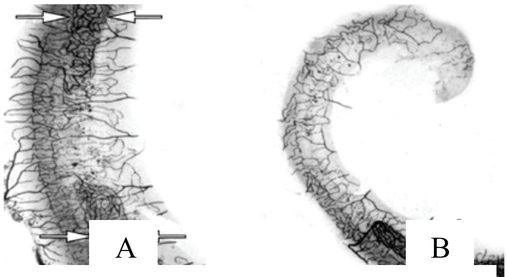

Fig. 2: Gerbil cochlea stria vascularis with focal (A) and diffuse (B) capillary loss

Sufficient cochlear blood flow is crucial for maintaining hair cell viability and preventing ischaemic deafness. Changes in the microcirculation of the cochlea have been considered an aetiological factor in many forms of hearing loss. Mouse models show strial atrophy, secondary to strial microvascular degeneration (24) and strial capillary loss is strongly associated with presbyacusis in aged gerbils (Fig. 2) (25) Histology of remaining capillaries shows thickened basement membranes, and strial vascular deposits of laminin and immunoglobulin. (26)

In gerbils, TNF-α has been shown to affect microvascular tone and reduce cochlear blood flow by reducing capillary diameter in the stria vascularis *in vivo*, forecasting ischaemic deafness. (27)

These studies present a compelling case for further investigating inflammatory effects on the microvascular frameworks of ageing human cochleae. The well-established association between ARHL and diabetes is at least suggestive of a microvascular commonality. Data from 3527 participants in the 30 year NHANES study demonstrate that diabetes is associated with increased odds of hearing impairment after controlling for age, sex, race, education, smoking and noise exposure. (28) Inflammatory vascular changes are implicated in conditions such as proliferative diabetic retinopathy, and inflammatory cytokines IL-6 and TNF-α are significantly higher in diabetics. (29) Importantly, in Bainbridge's study of the association between high frequency hearing loss and diabetes, the effect is attenuated when controlled for CRP. (30)

Accessing cochlear blood vessels in otherwise healthy adults is challenging. However, diabetic cochlear microangiopathy has been examined at autopsy. In diabetics vs controls, basilar membranes of strial capillaries were thickened and small vessels narrowed and irregular (31). The Blue Mountains Hearing study established an association between ARHL and retinal changes including arteriolar narrowing, haemorrhages, micro aneurysms and A-V nicking in women. (32) These studies raise a question about common vascular pathways of disease that will be addressed in ASP-HEARING with simultaneous repeated measures of hearing acuity and retinal vessels. It should also be acknowledged that auditory brainstem vulnerability to microvascular changes may be associated with neural presbyacusis. Apolipoprotein (APO ε4) genotype, a major risk factor for vascular disease, has been associated with a greater degree of hearing loss at the age of 85 years, in the Leiden 85 population study. (16)

ASPIRIN EFFECT ON MICROVESSELS AND INFLAMMATION

Aspirin is an important therapy in vascular disease states and its use as a secondary prevention tool is widely accepted in both cardiovascular and neurovascular disease. A collaborative meta-analysis of primary and secondary prevention trials demonstrated an absolute reduction in vascular events, especially in those who already have occlusive vascular disease. The nett benefit in the primary prevention setting was less clear but still notable for a 12% proportional reduction in serious vascular events. (33) Aspirin exerts the majority of its anti-inflammatory effects by disrupting prostanoid synthesis, and beneficial effects are directly related to platelet aggregation inhibition. Growing evidence suggests aspirin also has a direct vascular effect. Regular aspirin users who also take an antihypertensive agent have been shown to have retinal arterioles wider than those of non-users or occasional-users, after adjusting for age, blood pressure, smoking, diabetes, and non-steroidal anti-inflammatory medication use. (32) Low/intermediate doses of aspirin have been reported to selectively prevent experimental diabetic microangiopathy. (34)

Aspirin and other non-selective non-steroidal anti-inflammatory drugs inhibit the enzyme cyclo-oxygenase (COX), which is a key enzyme in the synthetic pathway for prostaglandins. During the biosynthesis of prostaglandins, various 'reactive oxygen species' (ROS) are produced and the expressions of genes coding for IL-1β, IL-6, TNF-α, COX-2 and iNOS are increased (35). Aspirin also stimulates the biosynthesis of other anti-inflammatory compounds referred to as 'aspirin-triggered 15-epi-lipoxins'. Production of these compounds is believed to underpin, at least in part, the drug's anti-inflammatory effect at low doses. (36).

Aspirin's action as an anti-inflammatory agent is well established and a reduction in inflammatory biomarkers has been demonstrated in a series of published trials. Not all studies have demonstrated suppression, possibly the result of the substantial natural variability of these biomarkers leading to a need for substantial numbers of subjects and precise analytical techniques. A reduction in inflammatory markers with administration of 100-300 mg aspirin has been demonstrated, as follows:

- In a randomised crossover trial of 40 patients with chronic stable angina, MCSF, IL-6, and CRP were all reduced after 6 weeks of 300mg aspirin compared to placebo ($P<0.05$ for all comparisons). (37)
- In 121 patients with metabolic syndrome, aspirin 100 and 300 mg/day for 2 weeks significantly decreased blood levels of CRP and thromboxane B2 compared with baseline. (38)
- Both CRP and TNF-α were significantly decreased by 100 mg aspirin daily at 7 and 30 days in a randomised trial of 115 patients with non-ST-segment elevation acute coronary syndrome. (39)
- In 16,297 adults ≥45 years old, combined aspirin and statin use was associated with a synergistically lower CRP concentration, especially among participants on aspirin >5 years.(40)

Another five trials did not demonstrate a reduction in inflammatory markers, although most were of small size and therefore potentially subject to a type 2 error. On the basis of these data, it appears that low dose aspirin may have a modest and variable but significant effect on suppressing biomarkers of inflammation, a potential cause of ARHL.

ASPIRIN AND HEARING LOSS

It is well accepted that high doses of aspirin (>1.5 g/day) are ototoxic, resulting in reversible hearing loss and tinnitus;(41) however high doses have also been shown to be protective against aminoglycoside-induced or cisplatin-induced hearing loss through anti-inflammatory and/or oxidative stress reduction mechanisms.(42) (43)

The dose of aspirin could be crucial in moderating its effect on hearing. Despite its anti-inflammatory potential, most studies have focussed on assessing aspirin as a risk factor *for* hearing loss. Of note, a cross sectional study of 26,917 men showed an increase in self-reported impairment associated with aspirin (dose and indication unspecified); whereas a prospective observational study of 62,261 women found self-reported hearing loss *did not* increase with aspirin. (44)

There are no previous studies specifically designed to evaluate low dose aspirin's effect on hearing; however *animal* studies are promising. A study on chinchillas concluded that aspirin protects against noise-related cochlea damage, particularly outer hair cell loss; but is restricted by a relatively narrow therapeutic window. (45) Treating gerbils with salicylate after toxic noise exposure reduces hair cell damage and provides immunohistological evidence of aspirin-mediated reduction of free radicals.(46) If cumulative noise exposure and ageing are synergistic (47), then aspirin's effect on noise toxicity may translate into an even greater effect on ARHL.

Human studies are limited; however a double blind RCT of 195 patients given gentamicin plus 3g aspirin or placebo, demonstrated that the incidence of gentamicin-induced hearing loss was significantly lower in the aspirin group.(42) A second small double blind RCT suggested that 1.5 g aspirin attenuated gentamicin-related ototoxicity. (48) These results are encouraging, but the effect of low dose aspirin on age-related hearing loss is still unexplored. We contend that low dose aspirin will reduce the progression of age-related hearing loss.

THE ASSOCIATION BETWEEN HEARING LOSS AND COGNITIVE DECLINE

The concurrence of cognitive decline and hearing deterioration should be expected due the association of each with ageing. However, further accounts demonstrate the association remains after controlling for any effects of age. (11) CI Lin demonstrated that hearing loss is associated with incident all-cause dementia after adjusting for age, sex, race, education, diabetes smoking and hypertension in an American cohort. (49) CI Lin's more recent study found that baseline hearing loss was independently associated with accelerated cognitive decline and incident cognitive impairment in older adults (Health ABC cohort). (4) Data from the Australian DYNOPTA study (n=4221) found probable cognitive impairment independently predicted faster rates of decline in hearing and incidence of cognitive impairment was associated with worse hearing thresholds. (11)

RESEARCH PLAN

ASP-HEARING is a three year RCT study that will leverage off the large-scale ASPREE trial. (50)

Relationship to ASPREE trial
ASPREE (ASPirin in Reducing Events in the Elderly) is a 5 year RCT comparing low-dose aspirin (daily slow release 100mg enteric coated) with placebo in 19,000 community-dwelling adults aged ≥70 years. The primary outcome is disability-free survival, in relation to physical disability and dementia. ASPREE participants are free of previous cardiovascular disease or stroke, dementia and other severe illness at trial entry. Although ASPREE has been funded by the US National Institutes of Health, the majority of the trial is in Australia with 16,500 participants and 2,500 in the US. At present, >13,700 Australian participants have been recruited. Recruitment will be completed by late 2014, with follow-up concluding by late 2017. Infrastructure, recruitment, data collection, follow-up and participant retention methods are well-established. Additional benefits for ASP-HEARING include access to comprehensive socio-demographic data, clinical history, significant clinical events (adjudicated endpoints), measures of activities of daily living, cognitive assessment, cardiovascular events, depression, other co-morbidities, medications and hospitalisations in the ASPREE cohort.

Recruitment: Participants are selected from general practices and undergo phone screening to assess suitability. Potential participants attend a baseline examination for testing and distribution of run-in (placebo) medication. Eligible participants attend further testing and are randomised to either aspirin or placebo at one month. The majority agree to provide blood and urine specimens which are stored for later biomarker and genetic analysis. ASPREE participants are telephoned regularly to support compliance. Evidence of major clinical events is collected from medical records and categorised by blinded outcomes committees.

Inclusion criteria: persons aged ≥70 years of age, who are English language competent, community dwelling, physically capable of regularly attending their GP, and providing informed consent.
Exclusion parameters include a history of: cardiovascular morbidity, serious intercurrent illness likely to cause death within the next 5 years, current or recurrent condition with a high risk of major bleeding, absolute contraindication or allergy to aspirin, current continuous use of aspirin or other anti-platelet drug or anti-coagulant, current participation in a clinical trial, history of dementia or a Modified Mini-Mental State Examination (3MS) score of ≤77/100, or a loss of ability to perform any one of the six Katz basic activities of daily living.

Study Medication: Randomised participants are given 12 months' supply of either aspirin or placebo. The study medication is active (100 mg of enteric coated acetylsalicylic acid) or placebo – both un-scored enteric coated white tablets of identical appearance. One tablet is to be taken daily, half an hour before other medications.

Measurements: Screening measurements include blood pressure (BP) and 3MS. After the run-in phase, measurements by the research staff at the baseline visit include: height, weight, family history of heart disease, stroke or dementia, smoking history, alcohol use, educational level, co-morbidity, concomitant medications, Center for Epidemiological Studies Depression Scale (CES D-10), Short-Form 12 (SF-12), Instrumental Activities of Daily Living (IADL), Hopkins Verbal Learning Test-Revised (HVLTR), COWAT-F (verbal fluency) and Symbol-Digit Modalities Test (SDMT), blood biochemistry (fasting total cholesterol, LSL, HDL, TG, haemoglobin, glucose and creatinine); Participants attend annually, but cognitive testing is only performed every two years after Year 1. We envisage most ASP-HEARING participants will have hearing tests conducted in a dedicated ASPREE trial bus without a sound proof booth. Sound attenuating ear muffs are considered satisfactory guards against ambient noise. Other studies have adopted this rationale. (51)

ASP-HEARING STUDY PLAN
1. RECRUITMENT: All newly enrolled ASPREE participants in 2014 will be invited at their baseline visit to enter ASP-HEARING, regardless of existing hearing impairment. Recruitment to

ASP-HEARING is during the final phase of ASPREE's recruitment in 2014, funded by Monash University, and it is anticipated that 1,800-2,100 of the remaining ASPREE trial recruits will consent to participate.

2. HEARING MEASURES: Hearing tests will be conducted without hearing aids. Any aids will be turned off and removed after testing instructions are heard and understood. Previously unknown hearing impairment will be advised to the participant's GP.

2.1 OTOSCOPY will be performed at the outset of the hearing evaluation. If the eardrum is occluded, the participant and their GP will be informed. Responsibility for management of any obstruction will rest with the GP. The participant will return for hearing reassessment following management of the obstruction, if this is initiated promptly within two months of randomisation.

2.2 AUDIOMETRY AIR and BONE CONDUCTION TESTS: In order to test the main hypothesis, hearing acuity will be tested at baseline and annually for the three years of ASP-HEARING. Pure Tone Audiometry: portable Interacoustics AD226 audiometers with ER3A inserts and sound-attenuating ear muffs, will be used to obtain air-conduction thresholds in both ears at standard octave frequencies (0.25, 0.5, 1, 2, 4, and 8 kHz). A pure tone average of air conduction thresholds at 0.5, 1, 2, and 4 kHz in the better ear will be the summary measure of hearing used in our analyses, as per the definition of hearing deficit used by the World Health Organization. (52) A bone conduction test will be performed on the better ear using two frequencies, 1 kHz and 4 kHz. This will enable distinction of age-associated sensorineural hearing loss from conductive loss – the latter irrelevant to this study. Audiometric equipment for baseline measures has been purchased with assistance from Monash University. Over the 3-year study period, even subtle changes in hearing threshold will be measureable in all participants, including any deterioration that is imposed on hearing impairment at baseline.

2.3 SPEECH IN NOISE TEST: The Listening in Spatialised Noise-Sentence Test (LiSN-S) (53) assesses the ability to understand speech in the presence of spatially-separated, competing talkers. A simple repetition response assesses a listener's speech reception threshold for target sentences within background noise. Speech discrimination is affected both by cochlear loss and by the presence of certain central auditory processing disorders, (54) and together with the pure tone results the LiSN-S will help this distinction to be made. This test will provide us with a vital measure of functional hearing, the Speech Reception Threshold in noise (SRTn).

2.4 HEARING HANDICAP AND NOISE EXPOSURE QUESTIONNAIRES: The 10 item Hearing Handicap Inventory for the Elderly (HHIE-S) (55) and the 11 item Baltimore Longitudinal Study of Aging Self-Reported Hearing and Noise Exposure Questionnaire (56) will be administered to provide information on hearing history, noise exposure, use of hearing aids and perceived hearing handicap. This will also assist in determining the extent to which previous noise exposure contributes to hearing thresholds.

3. INFLAMMATORY BIOMARKERS: To test subsidiary hypothesis 1, inflammatory biomarkers IL-6, CRP and TNF-α will be measured in blood samples taken at baseline and change in these biomarker levels will be determined from levels measured in 3-year bloods collected by the ASPREE Healthy Ageing Biobank.

4. RETINAL VESSEL IMAGING (RVI): To test subsidiary hypothesis 2, retinal photography will be used to measure retinal vessel diameters, branching and vessel wall opacity and tortuosity. We are seeking evidence of microvascular changes which may be a causal factor in presbyacusis. RVI provides a convenient and accessible measure of blood vessel change. ASP-HEARING will take advantage of RVI equipment currently used by ASPREE's Age Related Macular Degeneration (AMD) sub-study.

5. COGNITIVE FUNCTION TESTS: To test subsidiary hypothesis 3, results from annual cognitive function assessments will allow determination of associations and causal relationships

through comparisons of changes over time (annually) in the key factors of hearing threshold, retinal vessel and cognition. ASP-HEARING will take advantage of the 3MS scores obtained by the parent trial, which are suitable measures of cognitive decline, to inform this hypothesis. Cognitive function is assessed at Years 0, 1, and 3 in the parent trial; therefore ASP-HEARING will require one additional single 3MS measure at Year 2 to complete the annual measures.

TIMELINE: ASP-HEARING commences recruitment in 2014.

Unshaded boxes indicate measures not currently covered by ASPREE or other sub studies

**ASP-HEARING STUDY**	**2014**	**2015**	**2016**	**2017**	**2018**
Audiometry [a] and Otoscopy	X	X	X	X	Data Analysis Papers Reports
Hearing Questionnaires [b]	X	X	X	X	
Retinal Vascular Imaging	X	X	X	X	
Cognitive Function Tests [c]	X	X	X	X	
Inflammatory Biomarkers	X			X	
**ASPREE PARENT TRIAL**					
Cognitive Function Tests [c]	X	X		X	
Demographics & Physical Measurements,[d] BP, Health Behaviours Cardiovascular Biomarkers,[e], Lifestyle[f]	X	X	X	X	
**ASPREE RETINAL/BIOBANK SUB STUDIES**					
Retinal Vascular Imaging	X			X	
Inflammatory Biomarkers	X			X	
Shading highlights data collection supported by the ASPREE trial					

[a]Pure Tone Audiometry air conduction & bone conduction; LISN-STEST [b] HHIE-S & BLSA; [c]3MS, HVLT-Revised, COWAT-F, SDMT; [d] First language, education, family history, co-morbidity, height, weight, waist; [e]Cholesterol, LDL-C, HDL-C, triglycerides, Hb, gluc. creatinine, urine albumin:creatinine ratio; [f] Physical activity, smoking history & alcohol use; SF-12, IADL

POWER AND SAMPLE SIZE CALCULATIONS: To address the primary hypothesis, 1800 participants provide 80% power to detect a difference in average hearing level/threshold (HL) change from a mean annual dB HL increase of +0.86 in the placebo group to a mean annual dB HL increase of +0.64 in the aspirin group (assuming SD of 1.64 dB HL for changes in HL over 3 years). This calculation is based on the background increase per annum of HL in Australians aged ≥75, with an average Pure Tone Audiometry 0.5, 1, 2, 4 kHz trajectory of +0.86 dB.(8)

STATISTICAL ANALYSIS: A linear mixed model with HL at years 0, 1, 2, and 3 as the repeatedly measured outcome will be used for all analyses equivalent to a model for follow-up HL measures that adjusts for baseline HL. (57) For the main hypothesis, the model will include an interaction term between treatment group (placebo/aspirin) and time since randomisation; the estimated interaction effect will summarise the evidence for/against this hypothesis. This model assumes a missing at random (MAR) mechanism for any unobserved HL values. Secondary analyses will include a sensitivity analysis of findings to the MAR assumption, enabling exploration of whether differential drop-out between groups during the follow-up period affects the estimates of treatment effect.

The same model structure will be used to assess evidence for the subsidiary hypotheses. Separate analyses will be undertaken for the inflammatory biomarkers: TNF-α, IL-6 and CRP, for the retinal microvascular change indicators (retinal arteriolar, venular calibre and focal arteriolar narrowing), and for cognitive impairment as measured by 3MS noting that each of these measures is on a continuous scale. For example, for TNF-α, the model will be extended to include the baseline TNF-α values and its corresponding parameters relating it to HL at baseline will assess evidence for cross-sectional association at baseline. For assessing association of biomarker changes with progression of HL, the model will be further extended to include the time-dependent TNF-α change from baseline as an explanatory variable. For assessing whether TNF-α mediates an aspirin-induced

slowing of HL, the model will be extended again to include an interaction between treatment group (placebo/aspirin) and the time-dependent TNF-α change from baseline.

ETHICS: Written informed consent will be obtained from all ASP-HEARING participants. ASPREE has ethical approval from the Human Research Ethics Committees of Monash University, the Royal Australian College of General Practitioners, University of Tasmania, University of Adelaide, Australian National University and ACT Health. ASP-HEARING will seek additional ethical approval.

FEASIBILITY: The feasibility of ASP-HEARING is assured by the following:

1. The ASPREE trial has funding secured from both an NHMRC seed grant and the NIH ($US50m) and the ASPREE Healthy Ageing Biobank has been funded by the CSIRO and the National Cancer Institute (USA).
2. Recruitment processes are established with successful recruitment of 13,700 participants into ASPREE to date, with recruitment of the remaining 2,800 participants continuing until late 2014, enabling ASP-HEARING to enrol and follow-up the necessary numbers of participants.
3. Other ancillary studies to ASPREE successfully utilise Retinal Vascular Imaging techniques (ENVISion and SNORE-ASA), and have proved attractive to ASPREE participants.
4. The CI-A is CI-A on SNORE-ASA, CIB on ENVISion, and a named investigator on ASPREE; AI McNeil is the Principal Investigator of ASPREE; CI Nelson, CI Wolfe and AI Reid are named investigators; and CI Woods is the Executive Officer of ASPREE – providing a pivotal link that ensures smooth implementation of ASP-HEARING.
5. ASP-HEARING will extend collaborations with the National Acoustic Laboratory, The HEARing Cochlear Research Centre, Johns Hopkins University and RUSH University – USA.
6. CI Dillon, CI Rance and CI Lin are internationally recognised experts in hearing research.
7. Many investigators for this proposal have long-standing successful collaborations including previous NHMRC-funded studies.
8. Significant cost benefits are filtered to ASP-HEARING from ASPREE. Access to all currently funded elements estimated savings of $640 per recruitment and $300 per annual visit.
9. Retinal cameras in ASPREE clinics and installed in specially outfitted clinic vehicles are available for use in this study, funded by another NHMRC sub-study of ASPREE focused on age-related macular degeneration and from philanthropic donations.
10. ASPREE has achieved recruitment success across metropolitan and regional areas of 4 States in south-eastern Australia by taking study staff and laboratories to the people. A primary prevention trial of this magnitude and complexity in community-dwelling older people would not be feasible without the research infrastructure developed for ASPREE; based primarily in general practices supported by customized research vehicles. Please see Table 1. For elements of ASP-HEARING already funded by ASPREE and related sub studies.

OUTCOMES AND SIGNIFICANCE

Hearing loss is a leading cause of disease burden in older Australians. It is a distressing, common and expensive reality of older age, reported by nearly two thirds of adults ≥70 years. Hearing loss has been associated with multiple co-morbidities, and of particular interest is the association with cognitive decline. Hearing loss is a significant barrier to the enjoyment of healthy, fulfilling and interactive senior years. Social ramifications include embarrassment and withdrawal, leading to isolation and functional decline. The net cost of suffering is in excess of $11 billion. ASP-HEARING will examine hearing threshold changes with and without low dose aspirin in a cohort of ~1800 community dwelling older Australians over a 3-year period. If our hypotheses are proven, then low dose aspirin has potential as the first inexpensive and simple primary care intervention to slow development or progression of age-related hearing loss. Even if our hypotheses are not proven, together with information from the established ASPREE cohort, ASP-HEARING will better understand the patterns of comorbidity associated with hearing loss; understand the relationship between ageing and hearing loss; and accurately model the hearing loss impacts in a large cohort.

ASP-HEARING presents a unique, cost effective opportunity to help inform the evidence-base of a significant health issue in the fastest growing sector of the Australian population.

1. Karpa M, Gopinath B, Beath K et al. Associations between hearing impairment and mortality risk in older persons. Ann. Epidemiol. 2010;20(6):452-9.
2. Access Economics. Listen Hear Australia: 2006: 91
3. Zhan W, Cruickshanks K, Klein B et al. Modifiable determinants of hearing impairment in adults. Preventive Medicine. 2011;53(4-5):338-42.
4. Lin F, Yaffe K, Xia J et al. Hearing loss and cognitive decline in older adults. JAMA Internal Medicine. 2013;173(4):293-9.
5. Lin F, Thorpe R, Gordon-Salant S et al. Hearing loss prevalence and risk factors among older adults in the United States. J. of Gerontol. Series A, Biol. Sci. & Med.Sci. 2011;66(5):582-90.
6. Agrawal Y, Platz E, Niparko J. Prevalence of hearing loss and differences by demographic characteristics among US adults.1999-2004. Arch. of Int. Med.2008;168(14):1522-30.
7. Wilson D, Walsh P, Sanchez L et al. The epidemiology of hearing impairment in an Australian adult population. International Journal of Epidemiology. 1999;28(2):247-52.
8. Kiely K, Gopinath B, Mitchell P et al. Evaluating a dichotomized measure of self-reported hearing loss against gold standard audiometry: Journal of Aging and Health. 2012;24(3):439-58.
9. Dalton D, Cruickshanks K, Klein B et al. The impact of hearing loss on quality of life in older adults. The Gerontologist. 2003;43(5):661-8.
10. Kiely K, Anstey K, Luszcz M. Dual sensory loss and depressive symptoms: the importance of hearing, daily functioning, and activity engagement. Frontiers in Human Neurosci. 2013;7:837.
11. Kiely K, Gopinath B, Mitchell P, et al. Cognitive, health, & sociodemographic predictors of longitudinal decline in hearing acuity among older adults. J Geron -Biol Sci. 2012;67:997-1003.
12. Gopinath B, Schneider J, McMahon C et al. Severity of age-related hearing loss is associated with impaired activities of daily living. Age and Ageing. 2012;41(2):195-200.
13. Grue E, Ranhoff A, Noro A et al. Vision and hearing impairments and their associations with falling. Scandinavian Journal of Caring Sciences. 2009;23(4):635-43.
14. Genther D, Frick K, Chen D et al. Association of hearing loss with hospitalization and burden of disease in older adults. JAMA.2013;309(22):2322-4.
15. Verschuur C, Dowell A, Syddall H et al. Markers of inflammatory status are associated with hearing threshold in older people. Age and Ageing. 2012;41(1):92-7.
16. Kurniawan C, Westendorp R, de Craen A et al. Gene dose of apolipoprotein E and age-related hearing loss. Neurobiology of Aging. 2012;33(9):2230.e7-.e12.
17. Stevens G, Brunskill E. Global hearing impairment prevalence. Eur J Pub Hlth 2013:23; 146-52.
18. Bruunsgaard H. The clinical impact of systemic low-level inflammation in elderly populations. Danish Medical Bulletin. 2006;53(3):285-309
19. Bruunsgaard H, Andersen K et al. A High Plasma Concentration of TNF-α is associated with dementia in centenarians. J of Geront. Series A: Bio. Sci., Med. Sci. 1999;54(7):M357-M64.
20. Fujioka M, Okano H. Pro-inflamm cytokines in noise-damaged cochlea. J Neur Res. 2006:83
21. So H, Kim H, Lee J-H et al. Cisplatin cytotoxicity of auditory cells requires secretions of pro-inflammatory cytokines via activation of ERK and NF- κ B. JAss.Res in Oto 2007;8:338-55.
22. Menardo J, Tang Y, Ladrech S et al. Oxidative stress, inflam'n & autophagic stress as key mechanisms of premature ARHL in SAMP8 mouse cochlea.Antiox.Red.Signaling. 2012:16;263
23. Nash S, Cruickshanks K, Klein R et al. Long term variability of inflammatory markers and assoc. factors in a population based cohort. J. of the Am. Geriatrics Society. 2013;61(8):1269-76
24. OhlemillerK.Mechanisms&genes in human strial presbycusis-animal models.Br Res. 2009;1277
25. Gates G, Mills J. Presbycusis. The Lancet. 2005;366(9491):1111-20.
26. Sakaguchi N, Spicer S, Thomopoulos G et al. Immunoglobulin deposition in thickened basement membranes of aging strial capillaries. Hearing Research. 1997;109(1):83-91.
27. Scherer E, Yang J, Canis M et al. TNF-α enhances microvascular tone and reduces blood flow in the cochlea via enhanced sphingosine-1-phosphate signaling. Stroke. 2010;41(11):2618-24
28. Cheng Y, Gregg E, Saaddine J et al.Three decade change in the prevalence of hearing impairment and its association with diabetes in the US. Prev. Medicine. 2009;49(5):360-4.

29. Gologorsky D, Thanos A, et al. Therapeutic interventions against inflammatory and angiogenic mediators in proliferative diabetic retinopathy. Mediators of Inflamm. 2012:2012;
30. Bainbridge K, Cheng Y Cowie C. Potential Mediators of Diabetes-Related Hearing Impairment in the U.S. Population: NHANES 1999-2004. Diabetes Care. 2010;33(4):811-6.
31. Wackym P, Linthicum F. Diabetes mellitus and hearing loss: clinical and histopathologic relationships. The American Journal of Otology. 1986;7(3):176-82
32. Liew G, Wong T, Mitchell P, et al. Retinal microvascular abnormalities and age-related hearing loss: the Blue Mountains hearing study. Ear and hearing. 2007;28(3):394-401
33. Antithrombotic Trialists Collaboration, Aspirin in the primary and secondary prevention of vascular disease. Lancet. 2009;373(5):1849-60
34. Sun W, Gerhardinger C, Dagher Z et al. Aspirin at low-intermediate concentrations protects retinal vessels in experimental diabetic retinopathy. Diabetes. 2005;54(12):3418-26
35. Chung H, Cesari M, et al. Molecular inflammation. Ageing Res. Rev. 2009;(8):18-30
36. Brancaleone V, Gobbetti T, Cenac N, et al. A vasculo-protective circuit centered on lipoxin A4 and aspirin-triggered 15-epi-lipoxin A4. Blood. 2013;122(4):608-617
37. Ikonomidis I, Andreotti F, Economou E et al. Increased proinflammatory cytokines in patients with chronic stable angina and their reduction by aspirin. Circulation. 1999;100(8):793-798
38. Gao X, Adhikari C, Peng L et al. Efficacy of different doses of aspirin in decreasing blood levels of inflammatory markers J. of Pharm. and Pharmacol. 2009;61(11):1505-10.
39. Chen Y, Xu F, Zhang Y et al. Effect of aspirin plus clopidogrel on inflammatory markers in patients with non-ST-segment elevation acute coronary syndrome. Chin.M J. 2006;119(1):32-36
40. Fisher M, Cushman M, Knappertz V et al. An assessment of the joint associations of aspirin and statin use with C-reactive protein concentration. American Heart Journal. 2008;156(1):106-11.
41. Day RO, Graham G, Bieri D et al. Concentration-response relationships for salicylate-induced ototoxicity in normal volunteers. Br J. Clin. Pharmacol. 1989;28(6):695-702.
42. Sha S, Qiu J, Schacht J.Aspirin to prevent gentamicin-induced HL. NEJM 2006 ; 354:1856-7
43. Mukherjea D, Rybak L, Sheehan K, et al. The design and screening of drugs to prevent acquired sensorineural hearing loss. Expert Opin Drug Discov. 2012;6(5):491-505.
44. Curhan S, Shargorodsky J, Eavey R, et al. Analgesic use and the risk of hearing loss in women. Am. J. of Epid. 2012;176(6):544-54.
45. Coleman J, Huang X, Jackson R, et al. Dosing study on the effectiveness of salicylate/N-acetylcysteine for prevention of noise-induced hearing loss. Noise and Health. 2010;12:159-18
46. Yamashita D, Jiang H, Le Prell C, et al. Post-exposure treatment attenuates noise-induced hearing loss. Neuroscience. 2005;134(2):633-42.
47. Sharon G, Liberman C. Noise-induced & ARHL interactions. J Ac Soc. 2006;119:3268.
48. Behnoud F, Davoudpur K, Goodarzi MT. Can aspirin protect or at least attenuate gentamicin ototoxicity in humans? Saudi Medical Journal. 2009;30(9):1165-1169
49. Lin F, Ferrucci L, Metter E et al. Hearing loss and cognition in the Baltimore Longitudinal Study of Aging. Neuropsychology. 2011;25(6):763-70.
50. Study design of ASPirin in Reducing Events in the Elderly (ASPREE): an RCT. Contemporary Clinical Trials.2013;36(2):555-564
51. Canadian Longitudinal Study of Aging: Physical Assessments, 2013
52. World Health Organisation: Deafness and hearing loss, 2013
53. Cameron S, Glyde H, Dillon H. LiSN-S Test. J of Am Acad Audiology. 2011;22(10):697-709.
54. Glyde H, Cameron S, Dillon H et al. The effects of hearing impairment and aging on spatial processing. Ear and hearing. 2013;34(1):15-28.
55. Ventry I, Weinstein B. Hearing Handicap Inventory Elderly. Ear& Hearing. 1982;3(3):128-134
56. Baltimore Longitudinal Study of Aging:USA 2013.
57. Liang K, Zeger S. Longitudinal Data Analysis of Continuous and Discrete Responses for Pre-Post Designs. Sankhyā: The Indian Journal of Statistics, Series B. 2000;62(1):134-48.
