# Supplementary material for: Low-Dose Aspirin and Progression of Age-Related Hearing Loss: A Secondary Analysis of the ASPREE Randomized Clinical Trial
Source: JAMA Netw Open. 2024 Jul 25;7(7):e2424373. doi: 10.1001/jamanetworkopen.2024.24373 (PMC11273233; doi:10.1001/jamanetworkopen.2024.24373)
Supplement: Supplement 2. — eTable 1. Changes in Mean Hearing Thresholds (dB) Between Baseline and Year 3, and Comparing Aspirin to Placebo Groups eTable 2. Sensitivity Analysis—Difference in Change in Mean Hearing Thresholds (dB) Between Baseline and Year 3, Comparing Aspirin to Placebo Groups eTable 3. Demographic Characteristics Comparing Those Included in the Main Analysis and Those Excluded Due to Their Year 3 Hearing Evaluation Being Conducted After the Trial Period Concluded eAppendix. Covariate Measures eReference [file jamanetwopen-e2424373-s002.pdf]

Supplementary Online Content

Clark DPQ, Zhou Z, Hussain SM, et al. Low-dose aspirin and progression of age-related hearing loss: a secondary analysis of the ASPREE randomized clinical trial. *JAMA Netw Open.* 2024;7(7):e2424373. doi:10.1001/jamanetworkopen.2024.24373

eTable 1. Changes in Mean Hearing Thresholds (dB) Between Baseline and Year 3, and Comparing Aspirin to Placebo Groups

eTable 2. Sensitivity Analysis—Difference in Change in Mean Hearing Thresholds (dB) Between Baseline and Year 3, Comparing Aspirin to Placebo Groups

eTable 3. Demographic Characteristics Comparing Those Included in the Main Analysis and Those Excluded Due to Their Year 3 Hearing Evaluation Being Conducted After the Trial Period Concluded

eAppendix. Covariate Measures

eReference

This supplementary material has been provided by the authors to give readers additional information about their work.

© 2024 Clark DPQ et al. *JAMA Network Open.*

eTable 1. Changes in Mean Hearing Thresholds (dB) Between Baseline and Year 3, and Comparing Aspirin to Placebo Groups

Outcomes	Mean change at Year 3 (95% CI)	p-value
**Pure tones frequencies**		
0.25 kHz	-0.5 (-2.4, 1.4)	0.61
0.5 kHz	-0.1 (-1.6, 1.5)	0.92
1 kHz	0.9 (-0.3, 2.1)	0.16
2 kHz	1.0 (-0.4, 2.3)	0.16
4 kHz	0.2 (-1.3, 1.7)	0.8
8 kHz	-0.2 (-2.5, 2.2)	0.87
4FA	0.31 (-0.72, 1.34)	0.55
**SRT (dB)**	-0.1 (-1.2, 1.0)	0.86

Abbreviations: CI = Confidence interval, **dB** = decibels, **4FA =** averaged dB pure tones 0.5 kHz to 4 kHz, **SRT=** Speech reception threshold.

© 2024 Clark DPQ et al. *JAMA Network Open.*

eTable 2. Sensitivity Analysis—Difference in Change in Mean Hearing Thresholds (dB) Between Baseline and Year 3, Comparing Aspirin to Placebo Groups

Included participants whose 3-year examination occurred at 3 or 6 months after the conclusion of the clinical trial.

Outcomes	Mean change at Year 3 (95% CI)	p-value
**Pure tone frequencies**		
4 kHz		
3 months post end of trial	-0.01 (-1.2, 1.1)	0.99
6 months post end of trial	0.1 (-0.6, 0.9)	0.77
4FA		
3 months post end of trial	0.1 (-1.5, 1.7)	0.89
6 months post end of trial	0.3 (-0.8, 1.3)	0.63
**SRT (dB)**		
3 months post end of trial	0.1 (-0.6, 0.9)	0.70
6 months post end of trial	0.2 (-0.3, 0.6)	0.54

Abbreviations: **CI** = Confidence interval, **dB** = decibels, **4FA =** averaged dB pure tones 0.5 kHz to 4 kHz, **SRT=** Speech reception threshold

eTable 3. Demographic Characteristics Comparing Those Included in the Main Analysis and Those Excluded Due to Their Year 3 Hearing Evaluation Being Conducted After the Trial Period Concluded

Subgroup	No. of participants	Included in main analysis	Excluded from main analysis

© 2024 Clark DPQ et al. *JAMA Network Open.*

		(n = 279)	(n = 983)
**Age- N (%)**			
≤ 75 y	863	188 (67%)	675 (69%)
> 75 y	399	91 (33%)	308 (31%)
**Sex- N (%)**			
Male	604	154 (55%)	450 (46%)
Female	658	125 (45%)	533 (54%)
**Alcohol use- N (%)**			
Current/Former	1,094	240 (86%)	854 (87%)
Never	168	39 (14%)	129 (13%)
**Smoking- N (%)**			
Current/Former	559	122 (44%)	437 (44%)
Never	703	157 (56%)	546 (56%)
**Frailty- N (%)**			
Not frail	850	194 (70%)	656 (67%)
Prefrail/ Frail	412	85 (30%)	327 (33%)
**Diabetes- N (%)**			
Yes	147	35 (13%)	112 (11%)
No	1,115	244 (87%)	871 (89%)
**eGFR- N (%)**			
<45 (mL/min/1.73m2)	30	5 (2%)	25 (3%)
≥45 (mL/min/1.73m2)	1,232	274 (98%)	958 (97%)
**Hypertension- N (%)**			
Yes	918	198 (71%)	720 (73%)
No	344	81 (29%)	263 (27%)
**Hearing aid use- N (%)**			
Yes	247	50 (18%)	197 (20%)
No	1,015	229 (82%)	786 (80%)
**Hearing loss severity- N (%)**			
Normal (<20dB)	378	86 (31%)	292 (30%)
Mild (20-34dB)	549	117 (42%)	432 (44%)
Moderate (35-49dB)	248	58 (21%)	190 (19%)
Moderately severe (50-64dB)	68	14 (5%)	54 (6%)
Severe-profound (65dB+)	12	2 (1%)	10 (1%)
**Loud noise exposure- N (%)**			
Yes	140	34 (12%)	106 (11%)
No	1,112	241 (88%)	871 (89%)

Abbreviations

eGFR = estimated glomerular filtration rate. dB = decibel

eAppendix. Covariate Measures

Diabetes is defined from self-report or fasting glucose ≥126mg/dL or on glucose-lower medications. Hypertension is defined as blood pressure ≥140/90 mmHg or on

© 2024 Clark DPQ et al. *JAMA Network Open.*

antihypertensive medications. ‘Frail’ included individuals with 3 or more criteria of the adapted Fried frailty criteria, including body weight, strength, exhaustion, walking speed and physical activity while ‘pre-frail’ was defined as having 1 or 2 criteria.[1] For measurement of fasting blood glucose, participants were required to fast overnight, and their blood samples were collected in a local clinic or pathology centre. Blood pressure for each participant was measured in the seated position after at least five minutes of rest using an automated oscillometric device with an occluding cuff of appropriate size for the upper arm circumference. Three separate and consecutive BP readings, 1 minute apart, were performed, and the mean of these measurements was recorded. Other variables were collected by questionnaire.

© 2024 Clark DPQ et al. *JAMA Network Open.*

eReference

1. Fried, L. P., Tangen, C. M., Walston, J., Newman, A. B., Hirsch, C., Gottdiener, J., Seeman, T., Tracy, R., Kop, W. J., Burke, G., McBurnie, M. A., & Cardiovascular Health Study Collaborative Research Group (2001). Frailty in older adults: evidence for a phenotype. *The journals of gerontology. Series A, Biological sciences and medical sciences*, *56*(3), M146–M156. https://doi.org/10.1093/gerona/56.3.m146

© 2024 Clark DPQ et al. *JAMA Network Open.*
