# Supplementary material for: Low-Dose Aspirin and Progression of Age-Related Hearing Loss: A Secondary Analysis of the ASPREE Randomized Clinical Trial
Source: JAMA Netw Open. 2024 Jul 25;7(7):e2424373. doi: 10.1001/jamanetworkopen.2024.24373 (PMC11273233; doi:10.1001/jamanetworkopen.2024.24373)
Supplement: Supplement 3. — Data Sharing Statement [file jamanetwopen-e2424373-s003.pdf]

Data Sharing Statement

Clark. Low-Dose Aspirin and Progression of Age-Related Hearing Loss. *JAMA Netw Open*. Published July 25, 2024. doi:10.1001/jamanetworkopen.2024.24373

Data
Data available: No
